# Movement Preparation and Bilateral Modulation of Beta Activity in Aging and Parkinson’s Disease

**DOI:** 10.1371/journal.pone.0114817

**Published:** 2015-01-30

**Authors:** Hadj Boumediene Meziane, Clara Moisello, Bernardo Perfetti, Svetlana Kvint, Ioannis Ugo Isaias, Angelo Quartarone, Alessandro Di Rocco, Maria Felice Ghilardi

**Affiliations:** 1 Dept. of Physiology, Pharmacology & Neuroscience, CUNY Medical School, New York, New York, United States of America; 2 Department of Neurology, University Hospital Würzburg, Josef-Schneider-Str. 11, 97080 Würzburg, Germany; 3 Department of Neurosciences, Psychiatry and Anaesthesiological Sciences, University of Messina, Messina, Italy; 4 Department of Neurology, Movement Disorders Center, NYU-Langone School of Medicine, New York, New York, United States of America; University of Rome, ITALY

## Abstract

In previous studies of young subjects performing a reaction-time reaching task, we found that faster reaction times are associated with increased suppression of beta power over primary sensorimotor areas just before target presentation. Here we ascertain whether such beta decrease similarly occurs in normally aging subjects and also in patients with Parkinson’s disease (PD), where deficits in movement execution and abnormalities of beta power are usually present. We found that in both groups, beta power decreased during the motor task in the electrodes over the two primary sensorimotor areas. However, before target presentation, beta decreases in PD were significantly smaller over the right than over the left areas, while they were symmetrical in controls. In both groups, functional connectivity between the two regions, measured with imaginary coherence, increased before the target appearance; however, in PD, it decreased immediately after, while in controls, it remained elevated throughout motor planning. As in previous studies with young subjects, the degree of beta power before target appearance correlated with reaction time. The values of coherence during motor planning, instead, correlated with movement time, peak velocity and acceleration. We conclude that planning of prompt and fast movements partially depends on coordinated beta activity of both sensorimotor areas, already at the time of target presentation. The delayed onset of beta decreases over the right region observed in PD is possibly related to a decreased functional connectivity between the two areas, and this might account for deficits in force programming, movement duration and velocity modulation.

## Introduction

Extensive work with electroencephalographic (EEG) scalp recordings has shown that, in normal subjects, power in the beta range (13–30 Hz) changes both during motor performance—with different types of paradigms and effectors—and during imagery [1,2,3,4,5,6]. In particular, in electrodes over the primary sensorimotor areas, beta power starts decreasing around movement onset and reaches floor values during movement execution (see [7] for a recent review). This decrease of beta power has been also termed event-related “desynchronization” (ERD) since it likely originates from decreased levels of synchronous activity in the underlying neural substrates [8]. The recent finding that lower levels of beta power before the target appearance correspond to shorter reaction times [9] further suggests that the faster is the disengagement from the beta “idle” state, the faster are target processing and movement planning. Therefore, the decrease of beta power in that temporal window, over a network that includes the primary sensorimotor areas, likely reflects the engagement of top-down mechanisms related to the attentional and working memory processes that are needed to promote fast and efficient responses [9,10,11].

Parkinson’s disease (PD) is associated with deficits of working memory and attention that are related to movement planning and execution [12,13,14]. Importantly, beta power seems to be altered in PD. In fact, recordings of local field potentials from the basal ganglia have consistently shown an increased background power in the beta range that is partially linked to disease duration and the severity of bradykinesia [15,16], although it can be suppressed by levodopa treatment or deep brain stimulation [15,17,18,19,20,21]. These results, together with recent MEG findings that beta desynchronization during movement is reduced in PD compared to controls [22], suggest that the mechanisms leading to suppression of cortical beta synchronization during movement planning are impaired in PD. Whether the PD deficit in cortical beta modulation, especially over the sensorimotor areas, corresponds to abnormalities in movement preparation processes remains to be determined. In addition, it is not known whether the two hemispheres play different roles, especially considering that the two motor cortices might participate in different aspects of motor control [23]. Indeed, scalp EEG studies with voluntary arm movements in PD have focused mostly on modulation of the mu rhythm and post-movement beta increases [24,25,26,27], leaving these questions unanswered. Thus, in the present work, we investigated the early changes of beta power activity preceding reaching movements performed with the right dominant hand, in a choice-reaction time task [9]. With high density (hd) EEG, we examined beta oscillations in the time window around the target presentation, in two areas centered on C3 and C4. With this approach we determined, first, the pattern of early changes of beta power over both sensorimotor areas and inter-hemispheric coherence in normally aging subjects; second, whether patients with PD show the same activity pattern as age-matched controls. Finally, we verified whether such EEG signatures are associated with reaction time and other kinematic characteristics in both controls and patients.

## Materials and Methods

### Subjects

*Fourteen patients with PD (mean age ± SD: 61.7 ± 9.8 years; range from 54 to 71 years; four women) and fourteen age-matched normal subjects (mean age ± SD: 62.3 ± 5.9 years; range from 54 to 70 years; five women) participated in the study. The patients had a diagnosis of “clinically probable” idiopathic PD and were treated with dopaminergic therapy. Hoehn-Yahr stage, UPDRS-III scores, disease duration and the clinically most affect side (determined by UPDRS-III scores) are reported in Table 1. Patients with predominantly left side signs had similar Hoehn-Yahr stage, UPDRS III scores, disease duration and therapy to those with predominantly right signs (all: p>0.2). In patients, testing occurred while they were on drug, after their morning dose. During the entire testing, which lasted for less than one hour, patients’ performance was stable without signs of wearing off or dyskinesiae. Subjects in the control group had no history of neurological or psychiatric disorders.* All patients and controls were right handed, as determined by the Edinburgh inventory [28], had normal or corrected vision and had a Mini-Mental State Examination score higher than 26.

**Table 1:** Patients’ characteristics.

	**Gender**	**Hoehn&Yahr Stage**	**UPDRS III Scores**	**PD Duration (yrs)**	Most Affected Side*
1	F	2	22	13	L
2	M	2	18	5	R
3	M	2	18	3	R
4	M	2	17	4	R
5	M	2	24	6	R
6	M	2	20	9	L
7	M	1	13	2	R
8	F	1	4	1	R
9	F	3	19	10	R
10	M	1	11	2	L
11	M	2	20	8	L
12	M	1	13	5	R
13	F	2	17	8	L
14	M	2	18	4	L

* as determined by the UPDRS scores

### Ethics Statement

The experiments were conducted with the approval of the Institutional Review Boards of the New York University and the City College of New York, according to the principles expressed in the Declaration of Helsinki. All participants signed a written informed consent form.

### Experimental setup and task

Subjects were outfitted with a 256-electrode cap for hd-EEG recordings, sat in front of a computer screen, and performed a simple reaching motor task, RAN, which has been used previously and described in detail elsewhere [9,29]. Briefly, they moved a cursor with their right hand on a horizontal digitizing tablet (sampling rate 200 Hz). They were asked to perform fast, uncorrected, straight out-and-back movements from a central starting point to one of eight radially arrayed targets (distance 4.0 cm, 45° degrees apart) displayed as circles (1 cm radius) on the screen. Targets were presented in a random order every 3 s in three “blocks” of 32 movements each. Each target presentation time was 400 ms. After each block, subjects rested for about a minute. As previously reported [12,29], several spatial and temporal indices were measured for each movement. In particular, we computed: reaction times (time from target appearance to movement onset); amplitudes of peak velocity and acceleration; movement time (time from movement onset to reversal); path length (from onset to reversal). Between-group comparisons for kinematic variables were performed with one-way ANOVA (alpha = 0.05).

### EEG recording and signal processing

Continuous EEG signals were acquired during the entire session with a 256-channel EEG Geodesic Netamps system (Electrical Geodesics Inc, Eugene, Oregon). The EEG signals were recorded with a sampling frequency of 1000 Hz and were subsequently down-sampled to 250 Hz and bandpass filtered between 0.5 Hz-80 Hz, with a notch filter centered at 60 Hz. Recording reference was at Cz (vertex of head) and impedances were kept below 50 KOhm. The electrodes in the outermost circumference (chin and neck) were excluded and analysis was performed on the remaining 183 electrodes. The EEG data were processed and analyzed by MATLAB-based custom scripts and the following toolboxes: EEGLAB [30] for data preprocessing including Independent Component Analysis (ICA), and FieldTrip [31] for time-frequency analysis and statistical comparisons.

### Preprocessing

Channels affected by bad scalp-electrode contact were visually identified and replaced with spherical spline interpolation. EEG was segmented into epochs aligned on the time of the target appearance and lasting from −2 to 2.5 s after the target appearance. Importantly, the choice of this long epoch was done in the preprocessing stage to allow for different analyses to be performed on the same dataset. For the analysis performed after preprocessing in this specific study, we selected the interval of interest from −600ms to +600ms with respect to target appearance. Epochs containing sporadic artifacts (abnormal tension bursts, cough or similar) were rejected by visual inspection. The number of epochs excluded was similar in the two groups (controls: 2.1 ± 3.5; PD: 1.6 ± 2.9; t-test: p = 0.64). Stereotypical artifacts, such as blinks, heartbeat, eye movements and muscle tension, were removed by Independent Component Analysis (ICA; [32,33]), based on a well-established procedure previously detailed [34], now extensively used in EEG analyses.

### Selection of Regions of Interest

The main aim of this work was to define the dynamic of beta power changes during movement planning in both normal subjects and patients with PD on electrodes overlaying the right and left sensorimotor areas, mainly represented by C3 and C4. The use of hd-EEG allowed us to identify, for each subject, the electrodes closer to C3 and C4 that had the lowest value of beta power during movement execution, with the constraint of it being posterior to Fz and anterior to Pz. Thus, for each subject, we defined the region of interest (ROI) as the peak electrode and its six immediate neighbors, one on the Left and the other on the Right of Cz. The parameters used for computation of the time course of beta power changes are described in the following section. For the purpose of ROIs identification, we computed time-frequency representations for each channel, as described in the next paragraph; for each subject, we averaged all the trials and selected the negative peak electrodes as described above.

### Time-Frequency Analysis

The time varying spectral content of the EEG data was estimated using a Short-Time Fourier transform with a Hanning window of variable width, fitting a fixed number of cycles of the analyzed frequency and a 16 ms step size. We analyzed frequencies from 13 to 30 Hz in steps of 1 Hz, using seven cycle windows. The power estimates were then averaged over the entire beta range and log-transformed. For each participant, beta power was then normalized by subtracting the average power of a baseline interval chosen from 600 to 300 ms before the target presentation. First, we determine whether, in the control group, normalized beta power around the time of target presentation was similar in the right and left electrodes, by comparing the power of the two ROIs in the time interval from 200 ms before target presentation until 200 ms after target presentation. We used paired t-tests for each 16-ms bin and corrected for multiple comparisons. We repeated the same analyses in the data obtained in patients with PD. Since in patients with PD we found significant ROI power asymmetry in the 200-ms interval before target presentation, we computed the differences between the two ROIs in that time interval in both groups. We then used unpaired t-tests to further explore differences between control and patient groups. Finally, to assess whether reaction time was related to the degree of beta changes, we computed Pearson correlation coefficients between reaction time and normalized beta power in the time interval showing significant differences in the previous analysis.

### Connectivity Analysis

We computed spectral coherency [35,36] as estimate of connectivity between the two ROIs in the beta range. Coherency between two signals is defined as the normalized cross-spectrum (for extended formulas, see, for example, [37,38]). The absolute value of coherency is often used to quantify the strength of functional connections. However, this measure makes no distinction between instantaneous and truly time-delayed correlation and is hence dominated by effects of volume conduction when applied to EEG data. For this reason, we decided to focus our analysis on the imaginary part of Coherency only (ImCoh), as suggested by a number of authors [37,39,40]. ImCoh is systematically different from zero only for nonzero phase lags. Computation of coherency is based on the same Short Time Fourier transform used for the power analysis described in the previous paragraph. Complex coherence values for each time point in the beta range were obtained as the normalized cross-spectrum and ImCoh was extracted by selecting the imaginary part of such estimates. ImCoh for each subject was then normalized by subtracting the average value of the baseline interval (the baseline period was the same as in the time-frequency analysis [−0.6 to −0.3s] before visual stimuli). Movement-related ImCoh changes of the two groups were then compared by nonparametric unpaired t-test with Bonferroni correction. We finally used Pearson correlation coefficients between movement time and coherence values in the time interval showing significant differences in the previous analysis.

## Results

### Kinematics

**Figure 1:**
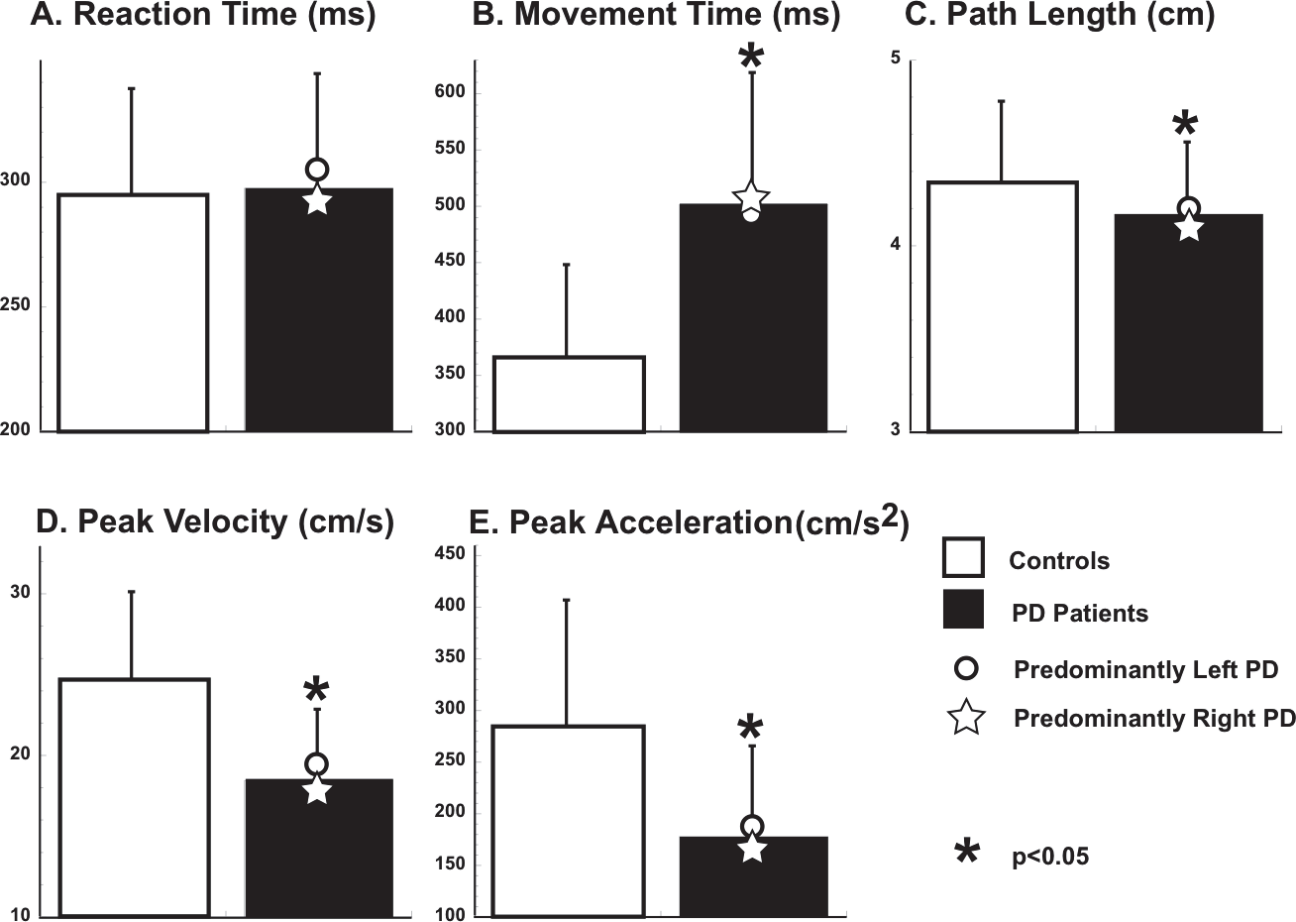
Kinematic measures. Means and standard deviations of reaction time (A), movement time (B), path length (C), peak velocity (D) and acceleration (E) are plotted for patients with PD (filled bars) and age-matched controls (empty bars). The stars and the empty circles represent the group mean of patients with clinically predominant right and left motor impairment, respectively.

In general, movement trajectories of patients and controls were essentially straight with sharp reversals and out-and-back overlapping strokes. In both groups, the velocity profiles of the outgoing movements were mostly single-peaked and, on average, bell-shaped with clear acceleration and deceleration phases. As expected for ballistic movements, peak velocity, peak acceleration and movement times were highly correlated both in combined and separate group analyses (all correlations: r always >0.80, p< 0.0001). Fig. 1 reports the means of several kinematic measurements, including reaction time and movement duration. On average, reaction time was similar in patients with PD and controls (F(1,24) = 0.03; p = 0.85). However, movement duration of patients was significantly longer (F(1,24) = 11.79, p = 0.002) and the peak of acceleration and velocity was reduced compared to controls (F(1,24) = 17.79, p = 0.0003; F(1,24) = 18.08, p = 0.0003). Similarly, path length was slightly shorter in PD patients compared to controls (F(1,24) = 5.54, p = 0.027). Importantly, there was no significant difference between the performance indices of patients with more severe right or left symptoms (p>0.3 for all comparisons, see Fig. 1).

#### In controls, beta power changes over motor areas are symmetrical

**Figure 2:**
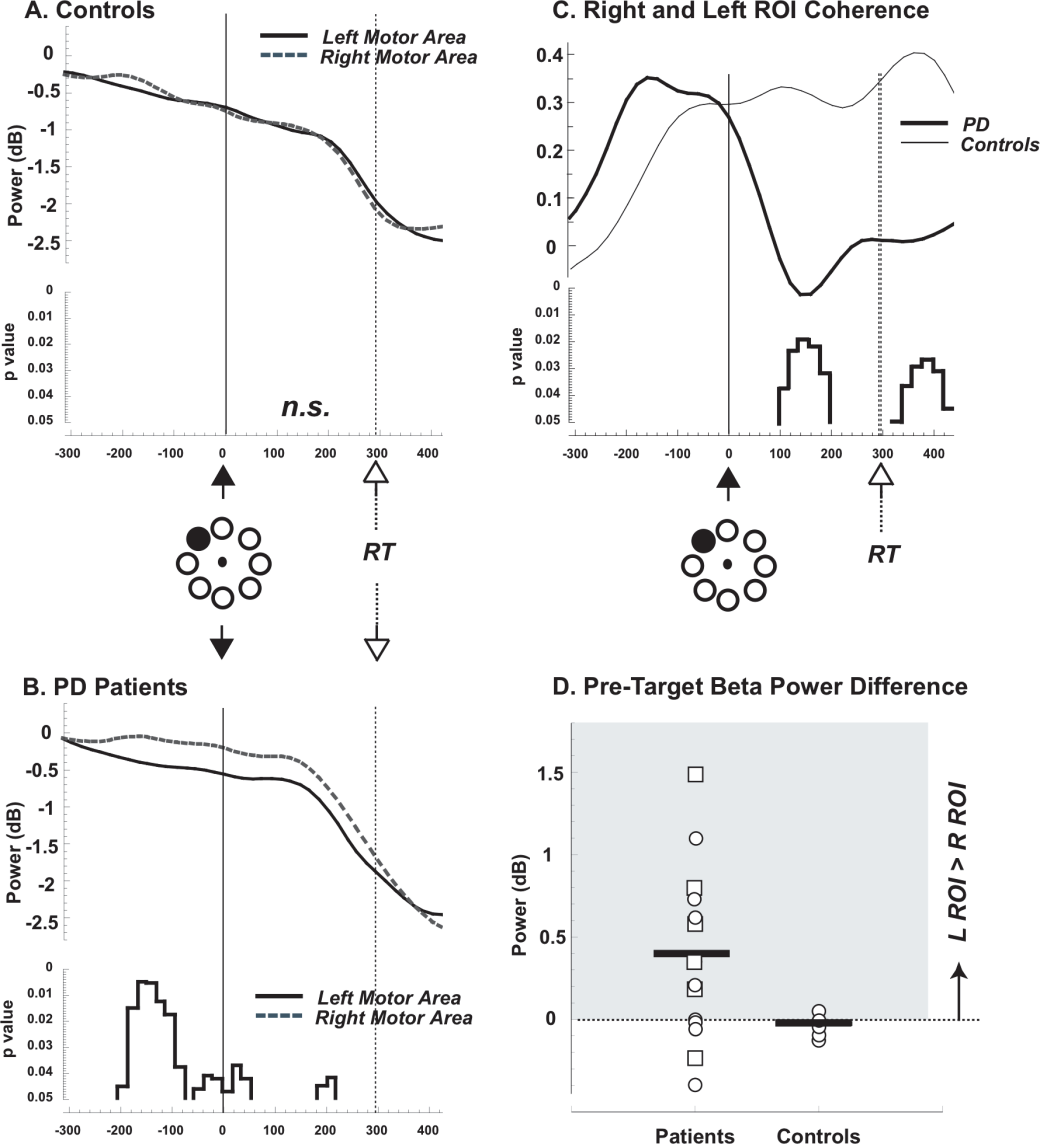
Beta power changes. A and B. Averages of the time courses of beta power changes in dB over the Left (solid line) and Right (dotted line) ROIs in controls (A) and patients with PD (B). The data were anchored at the time of target appearance (0 ms, filled arrows and thin solid lines). The empty arrows and thin dotted lines indicate the average time of movement onset (i.e., reaction time, RT) for each group. Negative time values on the X-axis represent the pre-target interval and from 0 to the empty arrow the reaction time period. Significant differences (p<0.05) between the two ROIs are indicated in the bottom part of each plot. C. Average of the normalized imaginary coherence in controls (thin line) and patients with PD (thick line). As in A and B, the data were anchored at the time of target appearance (0 ms, filled arrows). The empty arrows and vertical dotted lines indicate the average RT for each group. Significant differences between the two groups (p<0.05, nonparametric unpaired t-test with Bonferroni correction) are indicated in the bottom part of the plot. D. Individual differences of beta power between the left and the right ROIs averaged over the 200 ms time-interval before target appearance are plotted for the PD patients (on the left) and the controls (on the right). The thick horizontal lines represent the average for each group. The circles and the squares in the PD group represent patients with clinically predominant right and left motor impairment, respectively.

EEG data of three control subjects were excluded from the analyses because of technical problems during the recording. To characterize the time courses of normalized beta power changes over the two ROIs, for each subject, we selected the six electrodes for each area where movement-related beta desynchronization was maximal, as described in the methods. We then computed the time courses of beta power changes in these two regions for each subject and averaged them across subjects. The average results for the control group are presented in Fig. 2A. Briefly, we found a decrease in beta power over both ROIs that started before the target appearance, reaching a minimum during movement execution. Importantly, the two ROIs had overlapping time course throughout the motor task without any significant difference (Fig. 2A, all bins p>0.05).

We then measured the connectivity in the beta range between the two motor ROIs during the motor task, using the imaginary part of coherence. As shown in Fig. 2C, we found that, compared to a baseline, imaginary coherence values increased starting about 200–300 ms before the target presentation and reached a plateau that remained stable during the reaction time period and for most of the movement duration.

#### In patients with PD beta power changes over motor areas are not symmetrical

In patients with PD, like in controls, the changes of beta power started before movement onset (Fig. 2B). However, electrodes over the Right Motor ROI showed smaller power decrease than the Left, especially before target presentation, in the 200 ms time-interval before target appearance (Fig. 2B, paired t-test: p<0.05). Importantly, average differences between the two areas in this time interval were significantly greater in PD patients than in controls (Fig. 2D; unpaired two-tailed t-Test: p = 0.016).

Similarly to controls, imaginary coherence in PD increased before target presentation. However, these values dropped immediately after the target presentation, thus reaching values that were significantly lower from the controls (p<0.05, Fig. 2C) both during the reaction time period (time interval of 100–200 ms after target presentation) and at the movement onset (time interval of 320–440 ms after target presentation; see Fig. 2C).

**Figure 3:**
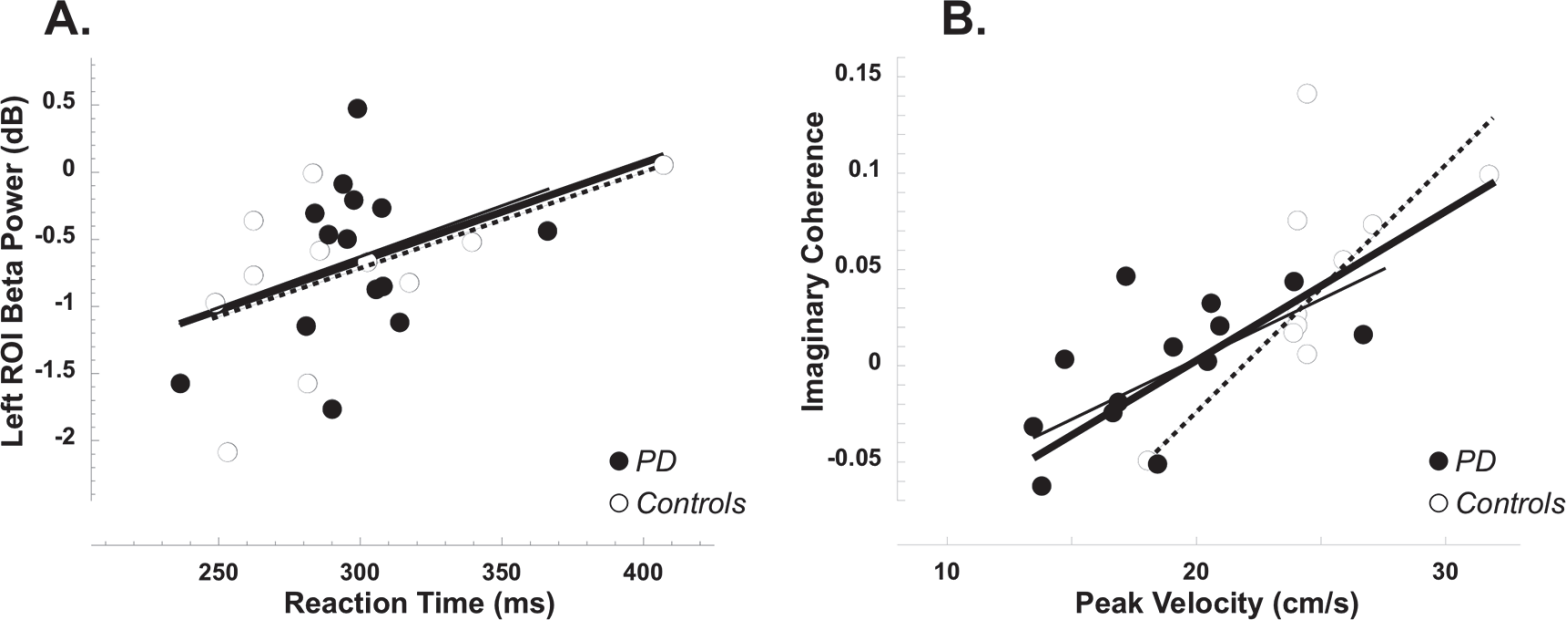
Correlative analyses. A. Reaction times (x axis) are plotted against the values of beta power over the left ROI averaged over the 200 ms before target presentation (y axis). B. The amplitudes of Peak Velocities (x axis) are plotted against the degree of imaginary coherence between the two sensorimotor ROIs in the period just after target presentation where significant differences between the two groups were found (y axis). In both plots, filled circles represent patients with PD and empty circles the controls; the thick lines represent the fitting lines on the entire data set (patient and control groups combined), while the thin lines are for the PD group and the dotted lines for the controls.

We then ascertained whether there was an effect of the clinically more affected side. Briefly, we found that beta power in the right ROI (averaged over the 200 ms before target presentation) had greater values when the left was the more affected side (0.14 ±0.09 dB) compared to a predominantly right-side impairment (−0.33 ± 0.10 dB; p = 0.003). For the Left ROI, there was only a trend toward significance in terms of clinically more affected side (left more affected: −0.21 ± 0.26 dB; right more affected: −0.67±0.16 dB, p = 0.07). However, affected side did not significantly influence both the average differences between the ROIs (p = 0.2; see squares versus circles in Fig. 3D) and the values of imaginary coherence (p always >0.2). Finally, the amount of medication (expressed in levodopa equivalents) was not correlated with either changes of beta power or values of normalized coherence (absolute r always <0.12; p>0.1).

#### Beta coherence between the two motor ROIs is important for efficient planning of fast movements

As previously found with a similar task in normal young subjects [9], we expected reaction times to be related to pre-target beta power. We thus performed correlative analyses between reaction times and the degree of beta power in the 200 ms before target presentation—where we found differences between the right and left ROIs in PD. We found some correlation between reaction times and levels of beta power over the left (both group combined: r = 0.44, p<0.02) and, although to a lesser extent, over the right (r = 0.36, p<0.05) ROIs, with shorter reaction times linked to greater beta power decreases. Correlations were still significant when the analysis was restricted to the control group (left: r = 0.54, p<0.05; right: r = 0.52, p<0.05), but not to the PD group (left: r = 0.34, p>0.05; right: r = 0.12, p>0.1; Fig. 3A) possibly due to a range effect. Finally, the values of normalized coherence just after target presentation (where significant differences between the two groups were found) significantly correlated with movement duration (both groups combined: r = −0.58, p<0.005; controls: r = −0.58; p<0.05; PD: r = −0.48; p<0.05) as well as with the amplitudes of peak velocity (both groups: r = 0.66, p<0.005; controls: r = 0.64, p<0.025; PD: r = 0.61; p = <0.02; Fig. 3B) and acceleration (both groups: r = 0.66, p<0.005; controls: r = 0.60, p<0.05; PD: r = 0.58; p = <0.025). In other words, the greater the coherence value, the faster the movement, indicating that efficient planning of fast movements requires coordinated activity of the two sensorimotor areas.

## Discussion

In this work, we characterized the changes of beta power that occur around the time of target presentation on the electrodes over the two sensorimotor areas during a choice-reaction time reaching task performed with the right dominant hand. In normal subjects, we found that the decrease of beta power before target presentation is symmetrical over the two hemispheres. Moreover, their functional connectivity, measured with imaginary coherence, increases before target presentation and remains high throughout the movement. In patients with PD, the beta power decreases earlier over the left hemisphere; coherence, on the other hand, increases with target presentation—similarly to controls-, but decreases afterwards, in the reaction time period. The results of correlative analyses further suggest that planning of prompt and fast movement is related, at least in part, to beta changes over both motor areas occurring before movement onset and that motor slowness in PD might be associated with a delayed onset of beta changes over the right motor area.

### The pattern of early beta power changes over the sensorimotor areas

Similarly to previous studies in young subjects [9,10,11], we found that the pre-target beta power over both motor areas was correlated with reaction time: the lower the power, the lower the reaction time. While early beta decreases over frontal and parieto-occipital regions [9] might be relevant to enhance alertness, attention, and readiness, the results of beta decrease over sensorimotor areas suggest that these areas must “emerge” from an idle state even before target specification. As suggested by the correlation with reaction times, an advanced preparation of sensorimotor areas would promote prompt and timely responses to task-relevant stimuli.

Our results in normal subjects further indicate that the level of beta desynchronization before target presentation is similar over the two motor ROIs, in agreement with a previous report ([4]), but not with other studies showing that decreases in pre-movement beta power are greater over the contralateral motor area. This discrepancy might stem from differences in the analytical approach, the type of task and the age of the subjects. In fact, we performed our analyses using as baseline the time from 600 to 300 ms before target, on individually selected ROIs (see methods). However, supplemental analyses using either the entire 3-second epoch as baseline or recordings only from the C3 and C4 electrodes yielded similar results (data not shown). Another source of the symmetrical power changes might be the complexity of the movements and task, as it has been shown that the likelihood of symmetrical bilateral activation increases with their complexity [7]. In fact, our study required reaching movements to unpredictable spatial locations, a task generally more complex than those producing preponderant contralateral desynchronization. In fact, those tasks required simple finger movements in response to visual or acoustic signals in pre-cued or predictable paradigms. Finally, age might have played a determinant role in the symmetrical pattern: virtually all the work on event-related desynchronization has been performed in young subjects (with an age range from 20–30 years). Indeed, the rather symmetrical early beta desynchronization in our controls might be partially attributed to age: in our previous work in normal young subjects tested with a similar task [9], beta decreases were slightly more prominent on the left hemisphere, contralaterally to the movement. There only are a few motor studies, all with simple finger movement tasks, directly addressing the effect of age on either alpha [41,42] or high alpha-low beta frequency ranges [43,44]. In general, those studies showed that, compared to the young, older subjects exhibit a rather symmetrical desynchronization pattern over the two motor areas in the alpha and beta range, suggesting that older subjects need a more extensive activation than the younger to achieve similar performance levels. Unlike normal subjects, the decrease of beta power around the time of target presentation in patients with PD was greater on the left than on the right electrodes. Although present in the majority of patients, this asymmetry was more prominent when the analysis was confined to patients with more severe symptoms on the left. Altogether, based upon these results, we speculate that in patients with PD, a more extensive recruitment–which should result in a symmetrical activation—might be hampered by an “altered status” or decreased timely access to relevant sensorimotor areas. The failure to do so might produce asymmetrical activation of sensorimotor areas and ultimately, might be the cause of slow movements, as suggested by our correlative analyses.

### Activity over the two motor areas must be coordinated for efficient movements

Our finding that imaginary coherence between the two areas increased at the time of target presentation further supports the notion that beta activity of the two motor areas is highly coordinated. Since it is not susceptible to volume conduction artifacts and “non-interacting sources cannot explain a nonvanishing imaginary coherency” [37,40], the imaginary part of coherence is a highly sensitive method and a good proof of a rise of coordinated activity between areas or sensors. Importantly, in control subjects, this increase reached a plateau during planning and this plateau value was sustained throughout movement duration. Other EEG studies, which did not take into account the temporal course, found that functional connectivity between the two motor areas generally increased in different frequency bands -including beta- during both bimanual and unimanual motor tasks of different sorts [45,46,47]. What are the possible anatomical bases underlying this functional connectivity? The corpus callosum provides anatomical connections between the two sensorimotor areas, as also suggested by the results of coherence studies in children and young subjects born with different degrees of acallosity [48,49]. However, other structures, such as the thalamus and the basal ganglia, could contribute to functional connectivity of the two sensorimotor areas. Indeed, the present findings that in patients with PD, the onset of beta desynchronization is asymmetrical and the values of imaginary coherence are not sustained during movement execution indicate that basal ganglia might play a role in functionally linking the two sensory-motor areas. Finally, since patients were tested in “ON” state in their regular medication schedule, the present results indicate that dopaminergic therapy cannot entirely restore coherence between the two areas, at least in this stage of the disease.

Although not symmetrical in temporal terms, involvement of both sensorimotor areas for unilateral reaching movements must occur at some point during any motor task. This has been invariably and widely shown in EEG and imaging studies in normal subjects [1,2,3,4,5,6] [50] and in patients with stroke [51,52]. Why would movements with the right dominant hand require the activity of both sensorimotor areas? The reasons could range from the inhibition of muscle activity of the non-moving side to postural adjustments needed by the production of aimed movement (for a review: [53]). In addition, the work of Sainburg and colleagues (see [23] for a review) indicates that the two sensorimotor areas might be involved in different aspects of motor control: the left dominant would be required for predictive or feedforward mechanisms, while the right non-dominant would largely control online or feedback processes. Therefore, the coordinated activity of both sensorimotor areas with reciprocal control is required for fast and efficient movements. Our present data further suggest that, in older subjects, for efficient motor planning and control, both areas must be engaged early on at the time of target presentation and that this bilateral engagement must be coordinated and sustained throughout the movement. Our findings further suggest that impaired functional connectivity between the two areas during the task might cause planning and control changes that are reflected in the kinematic characteristics. In fact, in our present results, lower coherence values corresponded to slower movements. In addition, in patients with PD, bradykinesia, i.e., low speed movements, seems to result from higher reliance on visual feedback when this is available during the movement, a problem related to online or feed-back movement control [29]. Based upon the present data, we can speculate that bradykinesia might stem from impaired functional connection between the two motor areas. Nevertheless, further studies are needed to prove this scenario right, to delineate the precise mechanisms as well as other areas and circuits involved in motor planning, execution and control, and to model the kinematic and behavioral consequences of failures of the system.

## Conclusions

Altogether, these data suggest that the planning of prompt and fast movements, at least in part, depends on coordinated beta activity of motor areas in both sides that should start at the time of target presentation. They further suggest that motor deficits in PD might be associated with a delayed onset of beta desynchronization in the right sensorimotor area compared to the left, possibly due to a decreased functional connectivity between the two areas. We can thus speculate that fast movements require the combined activity of the two areas and that the basal ganglia are involved in some measure in orchestrating this combined activity.

## References

[1] Alegre M, Labarga A, Gurtubay IG, Iriarte J, Malanda A, et al. (2003) Movement-related changes in cortical oscillatory activity in ballistic, sustained and negative movements. Exp Brain Res 148: 17–25. 10.1007/s00221-002-1255-x 12478393

[2] Doyle LM, Yarrow K, Brown P (2005) Lateralization of event-related beta desynchronization in the EEG during pre-cued reaction time tasks. Clin Neurophysiol 116: 1879–1888. 10.1016/j.clinph.2005.03.017 15979401

[3] Leocani L, Toro C, Zhuang P, Gerloff C, Hallett M (2001) Event-related desynchronization in reaction time paradigms: a comparison with event-related potentials and corticospinal excitability. Clin Neurophysiol 112: 923–930. 10.1016/S1388-2457(01)00530-2 11336910

[4] Salmelin R, Forss N, Knuutila J, Hari R (1995) Bilateral activation of the human somatomotor cortex by distal hand movements. Electroencephalogr Clin Neurophysiol 95: 444–452. 10.1016/0013-4694(95)00193-X 8536573

[5] Tombini M, Zappasodi F, Zollo L, Pellegrino G, Cavallo G, et al. (2009) Brain activity preceding a 2D manual catching task. Neuroimage 47: 1735–1746. 10.1016/j.neuroimage.2009.04.046 19389476

[6] Zaepffel M, Trachel R, Kilavik BE, Brochier T (2013) Modulations of EEG beta power during planning and execution of grasping movements. PLoS One 8: e60060 10.1371/journal.pone.0060060 23555884 PMC3605373

[7] Kilavik BE, Zaepffel M, Brovelli A, MacKay WA, Riehle A (2013) The ups and downs of beta oscillations in sensorimotor cortex. Exp Neurol 245: 15–26. 10.1016/j.expneurol.2012.09.014 23022918

[8] Neuper C, Pfurtscheller G (2001) Event-related dynamics of cortical rhythms: frequency-specific features and functional correlates. Int J Psychophysiol 43: 41–58. 10.1016/S0167-8760(01)00178-7 11742684

[9] Perfetti B, Moisello C, Landsness EC, Kvint S, Pruski A, et al. (2011) Temporal evolution of oscillatory activity predicts performance in a choice-reaction time reaching task. J Neurophysiol 105: 18–27. 10.1152/jn.00778.2010 21047934 PMC3023373

[10] Hanslmayr S, Aslan A, Staudigl T, Klimesch W, Herrmann CS, et al. (2007) Prestimulus oscillations predict visual perception performance between and within subjects. Neuroimage 37: 1465–1473. 10.1016/j.neuroimage.2007.07.011 17706433

[11] Zhang Y, Wang X, Bressler SL, Chen Y, Ding M (2008) Prestimulus cortical activity is correlated with speed of visuomotor processing. J Cogn Neurosci 20: 1915–1925. 10.1162/jocn.2008.20132 18370597

[12] Ghilardi MF, Eidelberg D, Silvestri G, Ghez C (2003) The differential effect of PD and normal aging on early explicit sequence learning. Neurology 60: 1313–1319. 10.1212/01.WNL.0000059545.69089.EE 12707435

[13] Kvint S, Bassiri B, Pruski A, Nia J, Nemet I, et al. (2011) Acquisition and retention of motor sequences: the effects of time of the day and sleep. Arch Ital Biol 149: 303–312. 10.4449/aib.v149i3.1244 22028091 PMC4321827

[14] Marinelli L, Perfetti B, Moisello C, Di Rocco A, Eidelberg D, et al. (2010) Increased reaction time predicts visual learning deficits in Parkinson′s disease. Mov Disord 25: 1498–1501. 10.1002/mds.23156 20568090 PMC3124249

[15] Brown P, Marsden CD (1999) Bradykinesia and impairment of EEG desynchronization in Parkinson′s disease. Mov Disord 14: 423–429. 10.1002/1531-8257(199905)14:3<423::AID-MDS1006>3.0.CO;2-V 10348464

[16] Tan H, Pogosyan A, Anzak A, Foltynie T, Limousin P, et al. (2013) Frequency specific activity in subthalamic nucleus correlates with hand bradykinesia in Parkinson′s disease. Exp Neurol 240: 122–129. 10.1016/j.expneurol.2012.11.011 23178580 PMC3605592

[17] Giannicola G, Marceglia S, Rossi L, Mrakic-Sposta S, Rampini P, et al. (2010) The effects of levodopa and ongoing deep brain stimulation on subthalamic beta oscillations in Parkinson′s disease. Exp Neurol 226: 120–127. 10.1016/j.expneurol.2010.08.011 20713047

[18] Kuhn AA, Doyle L, Pogosyan A, Yarrow K, Kupsch A, et al. (2006) Modulation of beta oscillations in the subthalamic area during motor imagery in Parkinson′s disease. Brain 129: 695–706. 10.1093/brain/awh715 16364953

[19] Kuhn AA, Kupsch A, Schneider GH, Brown P (2006) Reduction in subthalamic 8–35 Hz oscillatory activity correlates with clinical improvement in Parkinson′s disease. Eur J Neurosci 23: 1956–1960. 10.1111/j.1460-9568.2006.04717.x 16623853

[20] Levy R, Ashby P, Hutchison WD, Lang AE, Lozano AM, et al. (2002) Dependence of subthalamic nucleus oscillations on movement and dopamine in Parkinson′s disease. Brain 125: 1196–1209. 10.1093/brain/awf128 12023310

[21] Priori A, Foffani G, Pesenti A, Tamma F, Bianchi AM, et al. (2004) Rhythm-specific pharmacological modulation of subthalamic activity in Parkinson′s disease. Exp Neurol 189: 369–379. 10.1016/j.expneurol.2004.06.001 15380487

[22] Heinrichs-Graham E, Wilson TW, Santamaria PM, Heithoff SK, Torres-Russotto D, et al. (2013) Neuromagnetic Evidence of Abnormal Movement-Related Beta Desynchronization in Parkinson′s Disease. Cereb Cortex. 10.1093/cercor/bht121 PMC4153806 23645717

[23] Mutha PK, Haaland KY, Sainburg RL (2013) Rethinking motor lateralization: specialized but complementary mechanisms for motor control of each arm. PLoS One 8: e58582 10.1371/journal.pone.0058582 23472210 PMC3589347

[24] Defebvre L, Bourriez JL, Derambure P, Duhamel A, Guieu JD, et al. (1998) Influence of chronic administration of L-DOPA on event-related desynchronization of mu rhythm preceding voluntary movement in Parkinson′s disease. Electroencephalogr Clin Neurophysiol 109: 161–167. 10.1016/S0924-980X(97)00085-4 9741807

[25] Labyt E, Cassim F, Devos D, Bourriez JL, Destee A, et al. (2005) Abnormal cortical mechanisms in voluntary muscle relaxation in de novo parkinsonian patients. J Clin Neurophysiol 22: 192–203. 15933492

[26] Magnani G, Cursi M, Leocani L, Volonte MA, Comi G (2002) Acute effects of L-dopa on event-related desynchronization in Parkinson′s disease. Neurol Sci 23: 91–97. 10.1007/s100720200033 12391492

[27] Magnani G, Cursi M, Leocani L, Volonte MA, Locatelli T, et al. (1998) Event-related desynchronization to contingent negative variation and self-paced movement paradigms in Parkinson′s disease. Mov Disord 13: 653–660. 10.1002/mds.870130408 9686770

[28] Oldfield RC (1971) The assessment and analysis of handedness: the Edinburgh inventory. Neuropsychologia 9: 97–113. 10.1016/0028-3932(71)90067-4 5146491

[29] Ghilardi MF, Alberoni M, Rossi M, Franceschi M, Mariani C, et al. (2000) Visual feedback has differential effects on reaching movements in Parkinson′s and Alzheimer’s disease. Brain Res 876: 112–123. 10.1016/S0006-8993(00)02635-4 10973599

[30] Delorme A, Makeig S (2004) EEGLAB: an open source toolbox for analysis of single-trial EEG dynamics including independent component analysis. J Neurosci Methods 134: 9–21. 10.1016/j.jneumeth.2003.10.009 15102499

[31] Oostenveld R, Fries P, Maris E, Schoffelen JM (2011) FieldTrip: Open source software for advanced analysis of MEG, EEG, and invasive electrophysiological data. Comput Intell Neurosci 2011: 156869 10.1155/2011/156869 21253357 PMC3021840

[32] Makeig S, Debener S, Onton J, Delorme A (2004) Mining event-related brain dynamics. Trends Cogn Sci 8: 204–210. 10.1016/j.tics.2004.03.008 15120678

[33] Onton J, Makeig S (2006) Information-based modeling of event-related brain dynamics. Prog Brain Res 159: 99–120. 10.1016/S0079-6123(06)59007-7 17071226

[34] Jung TP, Makeig S, Humphries C, Lee TW, McKeown MJ, et al. (2000) Removing electroencephalographic artifacts by blind source separation. Psychophysiology 37: 163–178. 10.1017/S0048577200980259 10731767

[35] Nunez PL, Silberstein RB, Shi Z, Carpenter MR, Srinivasan R, et al. (1999) EEG coherency II: experimental comparisons of multiple measures. Clin Neurophysiol 110: 469–486. 10.1016/S1388-2457(98)00043-1 10363771

[36] Nunez PL, Srinivasan R, Westdorp AF, Wijesinghe RS, Tucker DM, et al. (1997) EEG coherency. I: Statistics, reference electrode, volume conduction, Laplacians, cortical imaging, and interpretation at multiple scales. Electroencephalogr Clin Neurophysiol 103: 499–515. 10.1016/S0013-4694(97)00066-7 9402881

[37] Haufe S, Nikulin VV, Muller KR, Nolte G (2013) A critical assessment of connectivity measures for EEG data: a simulation study. Neuroimage 64: 120–133. 10.1016/j.neuroimage.2012.09.036 23006806

[38] Formaggio E, Storti SF, Boscolo Galazzo I, Gandolfi M, Geroin C, et al. Time-Frequency Modulation of ERD and EEG Coherence in Robot-Assisted Hand Performance. Brain Topogr. 10.1007/s10548-014-0372-8 24838817

[39] Freyer F, Reinacher M, Nolte G, Dinse HR, Ritter P (2012) Repetitive tactile stimulation changes resting-state functional connectivity-implications for treatment of sensorimotor decline. Front Hum Neurosci 6: 144 10.3389/fnhum.2012.00144 22654748 PMC3358755

[40] Nolte G, Bai O, Wheaton L, Mari Z, Vorbach S, et al. (2004) Identifying true brain interaction from EEG data using the imaginary part of coherency. Clin Neurophysiol 115: 2292–2307. 10.1016/j.clinph.2004.04.029 15351371

[41] Babiloni C, Babiloni F, Carducci F, Cappa SF, Cincotti F, et al. (2004) Human cortical rhythms during visual delayed choice reaction time tasks. A high-resolution EEG study on normal aging. Behav Brain Res 153: 261–271. 10.1016/j.bbr.2003.12.012 15219728

[42] Derambure P, Defebvre L, Dujardin K, Bourriez JL, Jacquesson JM, et al. (1993) Effect of aging on the spatio-temporal pattern of event-related desynchronization during a voluntary movement. Electroencephalogr Clin Neurophysiol 89: 197–203. 10.1016/0168-5597(93)90133-A 7686852

[43] Sailer A, Dichgans J, Gerloff C (2000) The influence of normal aging on the cortical processing of a simple motor task. Neurology 55: 979–985. 10.1212/WNL.55.7.979 11061255

[44] Vallesi A, McIntosh AR, Kovacevic N, Chan SC, Stuss DT (2010) Age effects on the asymmetry of the motor system: evidence from cortical oscillatory activity. Biol Psychol 85: 213–218. 10.1016/j.biopsycho.2010.07.003 20637259

[45] Gross J, Pollok B, Dirks M, Timmermann L, Butz M, et al. (2005) Task-dependent oscillations during unimanual and bimanual movements in the human primary motor cortex and SMA studied with magnetoencephalography. Neuroimage 26: 91–98. 10.1016/j.neuroimage.2005.01.025 15862209

[46] Manganotti P, Gerloff C, Toro C, Katsuta H, Sadato N, et al. (1998) Task-related coherence and task-related spectral power changes during sequential finger movements. Electroencephalogr Clin Neurophysiol 109: 50–62. 10.1016/S0924-980X(97)00074-X 11003064

[47] Mima T, Matsuoka T, Hallett M (2000) Functional coupling of human right and left cortical motor areas demonstrated with partial coherence analysis. Neurosci Lett 287: 93–96. 10.1016/S0304-3940(00)01165-4 10854720

[48] Koeda T, Knyazeva M, Njiokiktjien C, Jonkman EJ, De Sonneville L, et al. (1995) The EEG in acallosal children. Coherence values in the resting state: left hemisphere compensatory mechanism? Electroencephalogr Clin Neurophysiol 95: 397–407. 10.1016/0013-4694(95)00171-9 8536568

[49] Montplaisir J, Nielsen T, Cote J, Boivin D, Rouleau I, et al. (1990) Interhemispheric EEG coherence before and after partial callosotomy. Clin Electroencephalogr 21: 42–47. 2297948 10.1177/155005949002100114

[50] Ghilardi M, Ghez C, Dhawan V, Moeller J, Mentis M, et al. (2000) Patterns of regional brain activation associated with different forms of motor learning. Brain Res 871: 127–145. 10.1016/S0006-8993(00)02365-9 10882792

[51] Mani S, Mutha PK, Przybyla A, Haaland KY, Good DC, et al. (2013) Contralesional motor deficits after unilateral stroke reflect hemisphere-specific control mechanisms. Brain 136: 1288–1303. 10.1093/brain/aws283 23358602 PMC3613707

[52] Schaefer SY, Mutha PK, Haaland KY, Sainburg RL (2012) Hemispheric specialization for movement control produces dissociable differences in online corrections after stroke. Cereb Cortex 22: 1407–1419. 10.1093/cercor/bhr237 21878488 PMC3357180

[53] Beaule V, Tremblay S, Theoret H (2012) Interhemispheric control of unilateral movement. Neural Plast 2012: 627816 10.1155/2012/627816 23304559 PMC3523159

